# CONTIGuator: a bacterial genomes finishing tool for structural insights on draft genomes

**DOI:** 10.1186/1751-0473-6-11

**Published:** 2011-06-21

**Authors:** Marco Galardini, Emanuele G Biondi, Marco Bazzicalupo, Alessio Mengoni

**Affiliations:** 1 Department of Evolutionary Biology, University of Firenze, via Romana 17, I-50125 Firenze, Italy; 2 Interdisciplinary Research Institute USR3078 - CNRS-University of Lille 1 - Université de Lille 2, Villenenuve d'Ascq, France

**Keywords:** Genomics, Genome finishing, Software, Structural genomics

## Abstract

Recent developments in sequencing technologies have given the opportunity to sequence many bacterial genomes with limited cost and labor, compared to previous techniques. However, a limiting step of genome sequencing is the finishing process, needed to infer the relative position of each contig and close sequencing gaps. An additional degree of complexity is given by bacterial species harboring more than one replicon, which are not contemplated by the currently available programs. The availability of a large number of bacterial genomes allows geneticists to use complete genomes (possibly from the same species) as templates for contigs mapping.

Here we present CONTIGuator, a software tool for contigs mapping over a reference genome which allows the visualization of a map of contigs, underlining loss and/or gain of genetic elements and permitting to finish multipartite genomes. The functionality of CONTIGuator was tested using four genomes, demonstrating its improved performances compared to currently available programs.

Our approach appears efficient, with a clear visualization, allowing the user to perform comparative structural genomics analysis on draft genomes. CONTIGuator is a Python script for Linux environments and can be used on normal desktop machines and can be downloaded from http://contiguator.sourceforge.net.

## Background

In the recent years, the dropping cost of sequencing technologies allowed biologist to easily widen the number of genomic sequences available for the scientific community, especially for bacterial species; moreover, the number of phylogenetically related genomes has also dramatically increased: the number of genera having more than 10 complete genomic sequences is 29 and interestingly, looking at the 12 species with more than 10 genomes fully sequenced, all of them belongs to bacteria (GOLD database [1], November 2010), pointing out the great value of closely related genomes in the so-called bacterial comparative genomics. However, looking at the ongoing or draft genomic projects, within 14 bacterial species with more than 50 running genomic projects, a lack of finishing efforts can be seen, suggesting that the problems encountered while closing a genome (even bacterial ones) are still time-consuming and cannot be easily automated. In fact, to close gaps of draft genomes a series of PCR reactions has to be designed in an iterative fashion.

To overcome this problems many programs have been recently developed, using an approach where all the contigs obtained by the first automated assembly run are mapped to a reference closed genome (usually inside the same species or as close as possible) and a series of PCR primers are designed to fill putative gaps existing between the contigs, in an iterative fashion. These programs are Projector2 [2], PGA4genomics [3], OSlay [4], ABACAS [5] and other algorithms present in genome annotation tools [6]; all of them could be used both for genomic finishing and, simply, to infer the relative position of the contigs, but none of them allows the user in finding divergent regions in the reference and the new genome, which could be helpful in performing preliminary structural analysis; moreover, in the case of a genome composed by many replicons, there is no automated procedure for multipartite genomic organization, avoiding to place one contig in more than one replicon.

In order to try to solve these problems and help genomic scientists in performing comparative structural analyses reducing the time-consuming PCR-based finishing process, we developed CONTIGuator, a script which combines the routines of one of mostly used tools, ABACAS, refining the results with a contig profiling viewable with the Artemis comparison tool (ACT) [7], in which the putative PCR products for the subsequent step of the finishing process are also shown.

## Implementation

**Figure 1:**
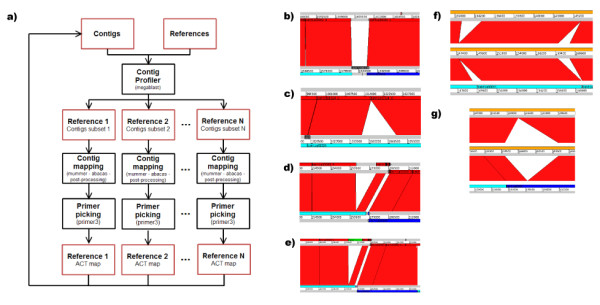
**CONTIGuator**. a) Program flowchart: the input contigs are mapped to the reference using the combination of Blast and MUMmer, generating a map (viewable with ACT), Primer3 provides a series of PCR primers that can be used to generate a new set of contigs, from which the process can be iterated again; b-c-d-e) ACT visualization: the reference genome is on top, pseudocontig on bottom; b) a putative primer placement over two near contigs; c) a section of no synteny in an otherwise fully syntenic contig; d) a region of the reference genome with no homolog in the mapped contig; e) the same region in d) highlighted, since it harbors a tblastn hit against the excluded contigs; f-g) verification of structural clues in ACT: the closed genome of *Sinorhizobium meliloti *BL225C is on top, the reference genome in the middle and the contigs on bottom.

The CONTIGuator pipeline is detailed in Figure 1a; the user provides a fasta file, containing all the contigs, and one or more fasta files with the replicon(s) of the reference genome: the first step of the analysis comprises a megablast run (the user can both have installed the new Blast+ [8] or the old Blast implementation [9]), which provides the contig-profiling. This step is useful for highlighting the parts of the reference and of the contigs that are homologous or divergent (some examples are given in Figure 1b, c and 1d); if the targeted genome is supposed to harbour more than one replicon, the program will ensure that each contig will be mapped only once. The intermediate output is then used as an input for the ABACAS perl script, one for each reference replicon, which performs a MUMmer [10] run (nucmer, delta-filter and show-tiling executables) and creates a first pseudocontig molecule. The first pseudocontig molecule is used and corrected by CONTIGuator to create the final pseudocontig molecule and the map. The contigs placed across the starting point of the molecule are splitted in order to generate a clearer map and the position of each contig is adjusted according to the blast output. This map will result in shorter gaps and therefore more PCR primers will be found in the subsequent step; as in ABACAS, the gaps between each contig is filled with Ns. The following step comprises the use of the Primer3 suite [11] to create a set of primers in order to fill the remaining gaps; the uniqueness of the primers is ensured by a nucmer scan over all the contigs given to the program. The last step is optional and needs the presence of the reference genome ptt files (usually available in the NCBI ftp repository) in order to use the protein sequences from non-homologous regions of the reference genomes for a tblastn run against the excluded contigs; the output will help the user to find those regions of the reference genome that may be still present in the draft genome, although with a lower level of homology and/or with a different arrangement.

The outputs of the program are divided in different directories (one for each replicon), containing the primers sequences (if the option was selected) and a series of input files for the Artemis comparison tool (ACT): "Reference.fsa", containing the reference sequence, "PseudoContig.fsa", containing the pseudocontig sequence, "PseudoContig.crunch", which is the Artemis comparison file. As soon as these files are loaded into ACT, the user can add the "ReferenceHits.tab" and the "PseudoContig.tab" entry file to show the blast hits on the reference genome and the position of the contigs in the pseudocontig molecule; if the primers were generated, the "PCRProducts.tab" entry file can be added to the pseudocontig molecule to see the putative PCR products. Finally, if the reference genomes ptt files were present, the user can add the "ReferenceProteinHits.tab" entry file to show the position of the tblastn hits on the reference genome.

## Results and discussion

CONTIGuator was tested on four closed genomes for which the first draft assembly was available: the *Brucella microti *[12] genome, which comprises two replicons of about 2 Mb and 1 Mb, respectively, whose draft assembly comprised 1539 contigs against the reference *Brucella melitensis *strain ATCC 23457 [13] genome, which also comprises two replicons of the same size; the 864 draft contigs from the *Yersinia enterocolitica subsp. palearctica *Y11 [14] genome against the 4.6 Mb long reference *Yersinia enterocolitica subsp. enterocolitica *8081 [15] genome, the 547 draft contigs from the *Lactococcus lactis subsp. lactis *KF147 [16] genome against the 2.6 Mb long reference *Lactococcus lactis subsp. lactis *Il1403 [17] genome and the 158 draft contigs from the *Sinorhizobium meliloti *BL225C [18] genome against the reference *S. meliloti *Rm1021 genome[19] genome, which comprises three replicons of about 3.7 Mb, 1.7 Mb and 1.4 Mb, respectively. A test run of CONTIGuator with the default parameters was performed and compared with ABACAS, PGA4genomics, OSlay and Projector2, as well as the primer picking for CONTIGuator, ABACAS and Projector2; the results of this test are presented in Table 1. With respect to ABACAS, CONTIGuator allowed the mapping of 257 kbp on average in addition to the other programs; since the pseudocontig map is corrected with the megablast output, there is a substantial gain in the number of PCR primers obtained (an average of 21 PCR pairs more), with respect to PGA4genomics, CONTIGuator allowing the mapping of 176 kbp more on average. CONTIGuator mapped 182 kbp less than OSlay; however OSlay does not produce a set of primers. Looking at the comparison with Projector2, CONTIGuator allowed the mapping of 1841 kbp more than the previous software: such a dramatic difference is mainly due to a putative malfunction of projector2 when analyzing the genome of *S. meliloti *BL225C, although CONTIGuator mapped more base pairs for the other genomes as well; however a compensation in the number of PCR primers generated by Projector2 (an average of 52 more PCR pairs) was observed, which could be due to the fact that both ABACAS and CONTIGuator ensure the uniqueness of the primers. Moreover, in the *B. microti *and *S. meliloti *tests, CONTIGuator ensured that no contig was assigned to more than one replicon, while with the other programs many contigs were assigned to more than one replicon. The ACT visualization, as shown in Figure 1(b, c, d, e, f, g), allows the user to highlight which portions of the contigs and of the reference genome are divergent and syntenic, allowing a first glance in the structural features of the new genome; in Figure 1f and 1g two examples of ACT visualizations generated by CONTIGuator were reported, showing experimental verification of the assembly prediction with the full genome sequence.

**Table 1:** Comparison between ABACAS and CONTIGuator performances over the four test genomes.

		*B. microti*	*Y. enterocolitica*	*L. lactis*	*S. meliloti*
**bp Mapped**	**CONTIGuator**	3291978	4164667	1912883	6350458
	**ABACAS**	3256036	3876600	1803685	5754197
	**PGA4genomics**	3267649	3090612	1761837	6897232
	**OSlay**	3286027	4236992	2020582	6906017
	**Projector2**	3271308	3160370	1661752	262344

**PCR pairs**	**CONTIGuator**	61	304	89	73
	**ABACAS**	49	255	78	62
	**Projector2**	71	278	367	20

**Gaps putatively closed**	**CONTIGuator**	58	171	63	39
	**ABACAS**	42	158	56	35
	**Projector2**	64	90	323	4

The base pairs derived from those contigs appearing in more than one replicon were divided by two.

Using the four complete genomes, we compared the three programs that are able to generate PCR primers (CONTIGuator, ABACAS, Projector2) in terms of how many gaps the generated set of PCR primers would putatively close (Table 1). We checked if the relative position of the contigs on the reference genome was the same when the contigs were mapped on the complete genome: the gap was considered as "putatively closed" when two contigs were mapped near each other on the reference genome and a PCR pair was designed between the two adjacent contigs. CONTIGuator was proven to perform better than ABACAS, since it lead to 10 extra putative gap closures than ABACAS; the main reason is the higher number of PCR pairs generated (12) was able to close more gaps. The other gaps, that ABACAS couldn't close, were due to a different relative placement of the contigs on the reference genome, a placement that in CONTIGuator was automatically corrected by the blast-based contig profiling. In comparison with Projector2, CONTIGuator by far closed more gaps in *Y. enteroclitica *and *S. meliloti*, slightly less for *B. microti *and less for *L. lactis*; it should be pointed out that the graphical output of projector2 lacks the contig profiler approach of CONTIGuator and therefore no detailed structural features can be seen prior to genome finishing. Moreover, as pointed out earlier, ABACAS (and therefore also CONTIGuator) ensures the uniqueness of the primer pairs generated, thus putatively removing any ambiguous reactions. An higher number of gaps closed (putatively in this simulation) could lead to a lower number of iterations (input contigs, CONTIGuator annealing, PCR reactions, new set of contigs) and therefore could strongly reduce the efforts in closing one genome. In the cases analyzed in this study, the apparent contradiction of more PCR pairs designed in the first run of the program, may lead to less time needed to close all the gaps in the draft genome.

## Conclusions

CONTIGuator is a powerful and fast algorithm for the bacterial genomes finishing process, providing a bigger PCR primers set able to close more gaps, and also giving clues on the relative position of the various contigs; moreover, CONTIGuator contigs profiling provides a high resolution map (viewable with the popular ACT program), highlighting regions of the reference genomes that are diverging from the assembled contigs. CONTIGuator indeed represents, before the end of the finishing phase, a powerful method for the investigation of the structural genomics based on draft genome sequences.

## Availability and requirements

CONTIGuator is a Python script for Linux environments and can be downloaded from http://contiguator.sourceforge.net, with a GNU/GPLv.3.0 license. The Python interpreter is needed with the addition of the BioPython package [20], as well as the Perl interpreter, a copy of ABACAS (available here: http://abacas.sourceforge.net/), as well as Blast+, MUMmer and primer3; all these programs must be reachable from the command line. The Artemis comparison tool is needed to view the output files. All the software packages were tested with their latest version, although no malfunction was reported with the older versions. The program performance was tested on a normal desktop machine (Linux Ubuntu 10.04, 4CPU Intel 2.50 GHz, 4 GB RAM) with run times in the order of a few minutes (about 6 minutes with primer picking and about 30 seconds without).

## Competing interests

The authors declare that they have no competing interests.

## Authors' contributions

MG wrote the script, tested it and contributed in drafting the manuscript. EGB, MB and AM conceived the idea and contributed in drafting the manuscript. All authors have given final approval of the version to be published.
